# Implementing nasal povidone-iodine decolonization to reduce infections in hemodialysis units: a qualitative assessment

**DOI:** 10.1017/ice.2024.83

**Published:** 2024-09

**Authors:** Kimberly C. Dukes, Stacey Hockett Sherlock, AM Racila, Loreen A. Herwaldt, Jesse Jacob, Anitha Vijayan, Joseph Kellogg, David Pegues, Pam C. Tolomeo, Jason Cobb, Mony Fraer, Susan C. Bleasdale, Melissa A. Ward, Brenna Lindsey, Marin L. Schweizer

**Affiliations:** 1 Department of Internal Medicine, Carver College of Medicine, University of Iowa, Iowa City, IA, USA; 2 Center for Access & Delivery Research & Evaluations (CADRE), Iowa City Veterans Affairs (VA) Health Care System (ICVAHCS), Iowa City, IA, USA; 3 College of Public Health, University of Iowa, Iowa City, IA, USA; 4 University of Iowa Hospitals & Clinics, Iowa City, IA, USA; 5 Department of Medicine, Emory University School of Medicine, Atlanta, GA, USA; 6 Department of Nephrology, Intermountain Health, Salt Lake City, UT, USA; 7 Division of Infectious Diseases, Hospital of the University of Pennsylvania, Philadelphia, PA, USA; 8 Division of Infectious Diseases, Perelman School of Medicine, Philadelphia, PA, USA; 9 Center for Clinical Epidemiology and Biostatistics, Perelman School of Medicine, University of Pennsylvania, Pennsylvania, PA, USA; 10 Renal Division, Emory University School of Medicine, Atlanta, GA, USA; 11 University of Illinois Hospital & Health Sciences System, Chicago, IL, USA; 12 University of Illinois at Chicago, Chicago, IL, USA; 13 Department of Medicine, School of Medicine and Public Health, University of Wisconsin, Madison, WI, USA

## Abstract

**Background:**

A substantial proportion of patients undergoing hemodialysis carry *Staphylococcus aureus* in their noses, and carriers are at increased risk of *S. aureus* bloodstream infections. Our pragmatic clinical trial implemented nasal povidone-iodine (PVI) decolonization for the prevention of bloodstream infections in the novel setting of hemodialysis units.

**Objective:**

We aimed to identify pragmatic strategies for implementing PVI decolonization among patients in outpatient hemodialysis units.

**Design:**

Qualitative descriptive study.

**Setting:**

Outpatient hemodialysis units affiliated with five US academic medical centers. Units varied in size, patient demographics, and geographic location.

**Interviewees:**

Sixty-six interviewees including nurses, hemodialysis technicians, research coordinators, and other personnel.

**Methods:**

We conducted interviews with personnel affiliated with all five academic medical centers and conducted thematic analysis of transcripts.

**Results:**

Hemodialysis units had varied success with patient recruitment, but interviewees reported that patients and healthcare personnel (HCP) found PVI decolonization acceptable and feasible. Leadership support, HCP engagement, and tailored patient-focused tools or strategies facilitated patient engagement and PVI implementation. Interviewees reported both patients and HCP sometimes underestimated patients’ infection risks and experienced infection-prevention fatigue. Other HCP barriers included limited staffing and poor staff engagement. Patient barriers included high health burdens, language barriers, memory issues, and lack of social support.

**Conclusion:**

Our qualitative study suggests that PVI decolonization would be acceptable to patients and clinical personnel, and implementation is feasible for outpatient hemodialysis units. Hemodialysis units could facilitate implementation by engaging unit leaders, patients and personnel, and developing education for patients about their infection risk.

## Introduction

Patients undergoing chronic hemodialysis have multiple risk factors for *Staphylococcus aureus* infections. Over a third of patients undergoing dialysis carry *S. aureus* in their nares, which increases their risk of access-related *S. aureus* bloodstream infections (BSI) by almost four-fold.^1^ In fact, their risk for methicillin-resistant *S. aureus* infections is 100 times that of the average person.^2^ The nares are the primary reservoir for *S. aureus* and from there they can contaminate patients’ skin^3^ and be spread between patients in a hemodialysis unit through contaminated furniture, equipment, or healthcare professionals’ (HCP) hands.^4^

The Society for Healthcare Epidemiology of America, Infectious Diseases Society of America, and Association for Professionals in Infection Control and Epidemiology recently recommended that dialysis units consider targeted or universal decolonization of patients on hemodialysis.^5^ Intranasal mupirocin decolonizes *S. aureus* carriage and can reduce infection rates for patients undergoing chronic hemodialysis.^3,6–10^ However, mupirocin decolonization can be difficult to implement and routine use may lead to mupirocin resistance.^7,8,10,11^ Intranasal povidone iodine (PVI) has been used to suppress *S. aureus* and to prevent infections in surgical settings and nursing homes.^12–16^ Compared with mupirocin, nasal PVI may be preferred given its price, patient preferences, and mechanism of action, which makes PVI resistance unlikely.^14–17^

We evaluated the implementation of nasal PVI in the novel setting of outpatient hemodialysis units during a pragmatic multicenter stepped-wedge cluster randomized trial that studied both PVI’s effectiveness and PVI implementation strategies. We previously described the study protocol.^18^ We aimed to evaluate HCP’s satisfaction with PVI decolonization and identify practical strategies that facilitate PVI decolonization in outpatient hemodialysis units.

## Methods

We conducted a qualitative descriptive study and evaluation to identify factors affecting implementation of PVI nasal decolonization during our trial (conducted from June 1, 2020 to May 31, 2023). We conducted interviews and performed thematic analysis on transcripts.

### Settings and processes

During this stepped-wedge trial, we implemented nasal PVI decolonization at 16 outpatient hemodialysis units affiliated with 5 US academic medical centers in 3 geographic regions.^18^ Participating units served a range of patient populations and included small rural units, mid-size urban and suburban units, and large urban units. The population of patients receiving dialysis in participating units ranged from 14 (two smallest units) to 203 (largest unit). Five units were operated by external hemodialysis providers contracted by the academic medical center. We translated patient-facing material into Spanish, French, and Mandarin Chinese. We asked patients to apply PVI to their nares before each hemodialysis session, at the unit, or at home.

### Data collection

The research team developed and piloted in-depth, semi-structured interview guides. We invited all research coordinators and staff at all hemodialysis units to participate. To increase participation, we also conducted site visits to seven hemodialysis units affiliated with three of the five partner universities. Qualitative experts conducted single and group interviews on virtual platforms, by phone, or in person. Interviewees included nurses, hemodialysis technicians, research coordinators, and other clinical and non-clinical personnel. We audio-recorded and transcribed interviews, and then imported transcripts into MAXQDA software.^19^

### Data analysis

We conducted thematic analysis of transcripts concurrent with data collection. The experienced qualitative team (KCD, SHS, AMR) collaboratively developed a thematic codebook that incorporated both *a priori* codes identified in the study’s design and inductive codes that emerged from interview data. First, we independently read a subset of transcripts (n = 4) to identify potential inductive codes, then met weekly using consensus to create and modify the codebook. To ensure coding fidelity given the variety of participant types, we coded seven transcripts using consensus, or negotiated, coding methodology.^20,21^ Subsequently we divided coding responsibilities and met weekly to resolve any coding ambiguities, and systematically documented discussions and rationales for codebook modifications. We periodically asked the principal investigator and site leaders to help us interpret emerging findings.

For this manuscript, we organized findings into four categories: trial context, patient-related factors, HCP-related factors, and strategies and tools to facilitate engagement of patients and HCPs. We chose to report trial-related barriers and facilitating factors to briefly provide context on PVI decolonization during the trial. We decided to present thematic findings about acceptability and engagement and perceptions of infection risk within sections divided by patient and HCP factors, because we wanted to identify elements common across trial units that could assist hemodialysis unit leaders interested in planning to implement PVI decolonization in their units.

The University of Iowa Institutional Review Board approved the study for three academic medical centers. The Institutional Review Boards at Emory University and University of Illinois Chicago approved the study for their sites.

## Results

We interviewed sixty-six participants. All interviewees worked in participating units or as research team members assisting participating units. No patients were interviewed. We report interviewee roles in Table 1. We provide exemplar quotes in Table 2.

**Table 1.** Summary of interviewees by role

Role	Number
Nurse	21
Dialysis technician	22
Administrative clinic staff	5
Other clinic staff (eg, dietitian, social work)	5
Unknown role clinic staff	5
Study coordinator	8
*sum*	*66*

**Table 2.** Selected exemplar quotes illuminating themes

Theme	Exemplar quotes
Awareness of patients’ infection risk	*For patients:* “… they don’t feel like they’re at risk until it [infection] personally happens to them.” (Site 5, Unit A, HCP) “…the younger patients are the ones that’s new to dialysis, they’re like, “I’m not gonna get sick.’” (Site 1, Unit A, HCP) “Some people tell me how clean they are and how they’re really good at washing their hands and keepin’ their house clean. …They never have to deal with infections.” (Site 4, RP) “Then, there’s another group who will say, “Well, I haven’t gotten *Staph aureus* yet, or I don’t know anybody who has had a *Staph* infection here, so why should I even do this?” (Site 2, RP) *For HCP:* “[One provider] just said, ‘Could kind of be a waste of time. I don’t see many patients who get bloodstream infections from *staph*, none of my patients.’ He said, ‘Only central catheter patients would be at risk, and I don’t have a lot of those on the list.’” (Site 4, RP) “…just fully educate the patient, and not only the patient, but the staff as well because we have a new wave of workers coming in and all that. … I’m working with experienced dialysis techs now, but ….” (Site 5, Unit A, HCP)
Acceptability and engagement	*For patients:* “For the people who have initially said yes, it’s mostly been like, ‘Anything I can do for my health. If this is gonna help me, and it’s not that much of a bother, why wouldn’t I?’” (Site 1, RP 2) “…a few who have said like, “Oh, I’m interested, but not right now because I just have too many other things going on.” Usually, the other things that are going on are health related. … Usually, pain is the most. They just don’t wanna think about something else.” (Site 1, RP 2) “Like I said, we have a lot of depression [among patients], so people are just--they don’t really care. Just getting by day by day.” (Site 3, Unit A, HCP) *For dialysis unit staff:* “[Patients] have built a trust with certain staff members. …. Once they [patients] find those relationships, and … who they trust, that information can be delivered. A lot of times it’s not the nurses. … ‘cause the nurses come and go, we have some [other] team members that have been here for years.” (Site 5, Unit A, HCP) *For leadership:* “When we started the intervention, there was pushback from the staff … the head of the dialysis center, …been really helpful … communicating to the staff about what the study is and what we’re doing.” (Site 2, RP) “…when [the] doctor is taking a round, [the] doctor can just brief them because they trust doctors too. Whatever the doctor told them, they will be like, “Okay. Dr. [X] told me about this. Give me that thing Dr. [X] was talking about.” (Site 1, Unit A, HCP)

Note. HCP, healthcare personnel; RP, research personnel.

### Trial context: summary of barriers and facilitating factors

Interviewees reported that hemodialysis units had varied success in recruiting patients and ensuring PVI was available to patients. Factors facilitating PVI use included educating patients and HCPs on infection risk; tailoring communication for specific patients; engaging unit staff, including non-clinical staff (eg, education specialists); ensuring that patients had access to PVI supplies and helping them apply PVI as needed; planning how to facilitate PVI use within available clinical space, and creating and using patient-friendly tools and strategies. Conversely, significant barriers included limited staffing, poor staff engagement, and perceived limited knowledge of patients’ infection risk among patients and some HCPs.

Interviewees identified important barriers to engaging HCP in PVI implementation during the trial, including high staff turnover, increased workload, and new infection-prevention protocols to prevent spread of SARS-CoV-2. Demonstrating these obstacles, interviewees at some sites reported staffing challenges that reduced their capacity to support the study. Interviewees also noted that clinical duties had increased, which reduced time for staff education. Consequently, some staff felt uninformed about PVI, and occasionally interviewees reported that they had actually learned about the PVI trial from participating patients. Additionally, interviewees perceived that PVI application was an add-on rather than an integral part of patient care. A further burden for staff was the variation in patient participation. Since patients could opt in or out of the study multiple times, HCP felt they did not have time to figure out patient participation while they were focusing on other tasks.

### Trial context: COVID-related barriers and infection-prevention fatigue

Interviewees reported that during the trial, the COVID-19 pandemic radically affected outpatient hemodialysis units, increasing HCP’s workload and stress. They pointed out that newly instituted infection-prevention practices—social distancing, universal masking, staggered patient arrivals, and waiting room restrictions—complicated both staff members’ workflow and their ability to integrate PVI application into their workflow. Consequently, both HCPs and patients experienced “infection prevention fatigue.” Furthermore, according to interviewees, both HCP and patients perceived that masking and social distancing could protect patients from other infections, which may have reduced their interest in or commitment to further infection-prevention activities like PVI decolonization.

### Patient-related factors

Throughout this section, we provide perspectives from interviewees (HCP and trial coordinators), who reported their own perceptions of patient attitudes and practices.

#### Acceptability and engagement

Interviewees reported that patients who used PVI generally found it acceptable. Interviewees did not report witnessing any major adverse or side effects and they noted that most patients were able to apply the PVI without assistance. However, interviewees described that some patients were unwilling to participate in research or were worried about iodine allergies. Study materials were available in four languages but some interviewees noted that the lack of other languages was a potential obstacle to engaging or educating patients about PVI use. Interviewees described patient-level challenges to PVI use. They reported that a small number of patients needed help applying PVI due to visual impairments or difficulty opening PVI bottles or applying PVI with the one hand they could use during dialysis. Interviewees also reported that some patients were too overwhelmed by health or life issues to add another task. Other patient-level barriers included depression, memory issues, lack of social support, and living situations that did not facilitate infection-prevention measures. Interviewees also reported that some patients expressed to them that nothing they did would improve their health and, thus, were unwilling to add infection-prevention practices. Some patients who used PVI at home told interviewees that using a calendar and incorporating PVI into their daily routines (eg, applying PVI when they did other personal hygiene activities like tooth brushing) was helpful.

#### Perceptions of infection risk

Interviewees reported that patients’ perceptions of their own infection risk could be either a facilitating factor or a barrier. For example, interviewees stated that patients who regularly used PVI (3 times per week) often noted their high infection risk and said they used PVI because they perceived direct benefits to themselves. Moreover, some patients told interviewees that they used PVI on non-dialysis days (eg, when shopping) and some requested access to PVI after the study ended. Conversely, interviewees reported that some patients were unaware of or unconcerned about their infection risk or were unaware that other patients had acquired serious infections and, thus, were unwilling to implement routine infection-prevention practices (eg, washing access sites). Some interviewees suggested that patient-centered conversations with trusted HCPs could improve patients’ understanding of their infection risk and the need for prevention measures, which could foster patients’ interest in using PVI.

### HCP-related factors

#### Acceptability and engagement

Interviewees generally found the PVI-related activities acceptable but did not feel the activities fit into their clinical workflow if they needed to first ascertain whether individual patients had consented. Clear communication about PVI’s potential benefit to patients and leadership support were reported to facilitate HCP engagement. According to interviewees, the level of intervention support varied across hemodialysis units, including some within the same academic institution. At some units, local clinical leaders (eg, nephrologists) strengthened implementation by expressing explicit support in staff meetings or discussing the trial directly with patients. However, interviewees at a few units reported that unit leaders were minimally involved in the study and that staff and patients who lacked knowledge about the intervention were not engaged.

Interviewees perceived that implementing PVI could be feasible and acceptable, particularly if integrated into routine practices for all patients and if HCPs were persuaded that PVI could help protect their patients from serious infections. Nevertheless, several interviewees identified workflow as a potential obstacle for some units and suggested that an alternative might be implementing PVI decolonization only for patients most at risk for access-related BSI.

#### Perceptions of infection risk

Interviewees noted that some HCPs thought their patients seldom had BSIs, and thus additional infection-prevention activities were unnecessary. Some interviewees suggested that they or their peers could also benefit from clear, evidence-based, recurring education about patients’ risks for access-related BSIs and potential benefits of PVI decolonization.

HCPs identified themselves as important advocates for infection prevention and appropriate, trusted partners in conversations with patients about infection risk and prevention. Some interviewees suggested that dietitians, education specialists, social workers, and clinic receptionists could encourage patients to use PVI or answer their questions and that dietitians and educators particularly could help patients develop strategies to integrate PVI into their routines.

### Strategies and tools to facilitate engagement of patients and HCP

Interviewees identified the following facilitating factors for PVI use on the unit, both during the trial or for wider clinical implementation: a designated accessible space where patients picked up their own PVI bottles and swabs or PVI delivery to patients at their chairs, and individual trash cans near patients’ chairs in which to dispose of used PVI and packaging. To support engagement of both patients and HCP, interviewees reported leadership support of PVI use, clear communication about infection risk and the potential role of decolonization, and deliberate and repeated messaging about PVI to HCP and patients in staff meetings, one-on-one discussions, and through videos and flyers.

Tools used during the study (Table 3) included signs and flyers; three-dimensional plastic noses to use while demonstrating PVI application; “take home” bags containing a month’s supply of PVI, tracking materials (eg, calendar or checklist), and instructions for PVI use; and instructive videos and flyers. Interviewees found study material acceptable. However, they recommended tailoring materials for different patient populations in the future. For example, interviewees also recommended any new educational material aimed at either HCPs or patients use simple, direct language; translation or interpretation; multiple formats; and images that reflected the unit’s patient population, specifically with respect to race and/or ethnicity. They also suggested developing additional videos or infographics discussing patients’ infection risk or sharing personal stories of patients who acquired serious infections.

**Table 3.** Tools used and purposes.

Purpose	Tools
Communication	Signs (for HCP and patients) Flyers (trifold, laminated) Video instructions Paper instructions Three-dimensional plastic noses (for demonstrating PVI application)
PVI delivery	Attractive boxes (for holding supplies at the unit for patients to pick up) “Take-home” bags (zip plastic and/or paper, to hold PVI supplies to take home) Paper calendars (for patient to track home PVI use)
Study delivery and patient consenting	Buttons with study logo (to identify coordinator or study staff) Sticky notes (to identify participating patients) Unofficial scripts to describe PVI to patients or answer common questions Excel sheets (to take notes about patients)
Building goodwill among HCP and patients	Study-branded pens Sugar-free candy

Note. HCP, healthcare professionals; PVI, povidone-iodine

## Discussion

Our interviews with staff in outpatient hemodialysis units suggest that PVI nasal decolonization to prevent *S. aureus* BSI among patients on hemodialysis could be feasible. However, we also identified numerous barriers to engaging HCPs and patients in our trial. Our interviewees reported that many patients found nasal PVI acceptable. Similarly, HCP felt that nasal PVI could be incorporated into clinical practice if hemodialysis units decided that routine *S. aureus* decolonization was a patient-safety goal.

Nevertheless, engaging patients in novel infection-prevention practices is difficult. Patients undergoing hemodialysis have high rates of depression and anxiety,^22–25^ which can reduce adherence.^26,27^ Jones *et al* found that hemodialysis processes create multiple stresses and that patients’ cognitive and physical well-being can fluctuate across the dialysis cycle.^25^ The interviewees in our study noted that obstacles included the overall high health burden for patients, depression, and cognitive issues. Units could consider how to support patients in integrating PVI decolonization into their routines to minimize patient burden.

Patient perspectives might also facilitate implementation. A scoping review of patient involvement in infection prevention and control concluded that HCPs and patients should collaborate to improve implementation of guidelines and associated interventions.^28^ More specific to the hemodialysis setting, several groups have found that patients undergoing hemodialysis have crucial perspectives on infection-prevention practices and their own roles.^29–33^ We did not interview patients. Future work should investigate what patients know about their infection risks and what they are willing to do for prevention. We should also test the efficacy of various engagement and educational strategies in different populations of patients on hemodialysis to help us develop more effective strategies for engaging patients in novel infection-prevention activities such as PVI nasal decolonization.

Similarly, HCPs also experience barriers to engaging in new infection-prevention practices. A large needs assessment conducted by Fitzgerald *et al* identified barriers to implementing infection prevention and control practices in hemodialysis units, including HCPs’ time constraints/high patient volumes, lack of motivation and knowledge, and low patient engagement.^34^ Our interviewees identified similar barriers in their units. Over the past 30 years, HCPs in dialysis units have reported experiencing stress and burnout.^35^ This has been exacerbated during the COVID-19 pandemic.^36^ Our study also found HCPs’ work stress reduced their capacity to take an active role in the trial, and thus work stress may impede implementation of new infection-prevention measures. However, McAlearney *et al* found that nurses’ perspectives and action could improve implementation of a central-line-associated BSI prevention initiative.^37^ This suggests that infection-prevention staff who wish to implement intranasal PVI to decrease *S. aureus* infections in hemodialysis settings must understand work processes in dialysis units and must work directly with unit staff to develop a protocol that works for staff and patients.

Multiple studies have shown that patients undergoing hemodialysis are at high risk for *S. aureus* infections.^1,2^ A study by Rha *et al* found that the risk of *S. aureus* BSI is also higher for non-Hispanic Black or African American patients and Hispanic or Latino patients than for white patients.^2^ Nevertheless, Pedersen *et al* recently found that most HCPs working in an academic medical center who responded to a survey were unaware that healthcare associated infection (HAI) rates vary by race and ethnicity.^38^ Interviewees in our study also did not discuss racial or ethnic differences in infection risk, suggesting there is an opportunity to improve understanding and develop interventions to reduce those disparities. In addition, interviewees reported that patients and some HCPs underestimated patients’ infection risk in general. In fact, some HCPs felt their patients were unlikely to acquire BSIs. This misunderstanding was a major barrier to implementing intranasal PVI decolonization. Thus, an early step in the integration of infection-prevention interventions like intranasal PVI into clinical practice, would be to increase HCPs’ and patients’ awareness of the patients’ infection risk in order to increase their willingness to adopt preventive measures.

This study has limitations. We implemented PVI decolonization as part of an implementation-effectiveness clinical trial testing whether it would reduce BSIs; however, the COVID-19 pandemic not only reduced hemodialysis units’ ability to actively implement the intervention but also increased other infection mitigation practices. While the 16 participating hemodialysis units included a wide range of patients and units, all were located in the US and other countries might experience different barriers. Additionally, implementation of a practice as part of a study can differ from implementation as clinical practice. In our trial, patients could consent or decline to participate, and HCPs were not required to deliver the intervention. Given the units’ challenges, research staff facilitated implementation. Thus, we may have missed some barriers or facilitating factors to implementing PVI decolonization as part of routine practice. We had limited access to HCP at some units and thus needed to elicit the local context from research coordinators, and we did not interview patients directly. Thus, assumption bias may affect our findings. Nevertheless, our findings were consistent across units.

While engaging HCP was difficult during the clinical trial, the results from our qualitative study suggest that both HCPs and patients would find PVI decolonization acceptable. Our results suggest that implementation of PVI decolonization could be feasible for outpatient hemodialysis units if it is supported by clinical leaders, HCPs are engaged, HCPs and patients understand the risk of infection and the role of decolonization in preventing infection, and PVI application is integrated into routine clinical care.

## Clinical trial information

ClinicalTrials.gov Identifier: NCT04210505, https://clinicaltrials.gov/.

## References

[1] Saxena AK , Panhotra BR , Venkateshappa CK , et al. The impact of nasal carriage of methicillin-resistant and methicillin-susceptible *Staphylococcus aureus* (MRSA & MSSA) on vascular access-related septicemia among patients with type-II diabetes on dialysis. Ren Fail 2002;24:763–777. 12472199 10.1081/jdi-120015679

[2] Rha B , See I , Dunham L , et al. Vital signs: health disparities in hemodialysis-associated *Staphylococcus aureus* bloodstream infections - United States, 2017–2020. MMWR Morb Mortal Wkly Rep 2023;72:153–159. 36757874 10.15585/mmwr.mm7206e1 PMC9925139

[3] Boelaert JR , Van Landuyt HW , Gordts BZ , De Baere YA , Messer SA , Herwaldt LA. Nasal and cutaneous carriage of *Staphylococcus aureus* in hemodialysis patients: the effect of nasal mupirocin. Infect Control Hosp Epidemiol 1996;17:809–811. 8985768 10.1086/647241

[4] Grothe C , Taminato M , Belasco A , Sesso R , Barbosa D. Prophylactic treatment of chronic renal disease in patients undergoing peritoneal dialysis and colonized by *Staphylococcus aureus*: a systematic review and meta-analysis. BMC Nephrol 2016;17:115–115. 27527505 10.1186/s12882-016-0329-0 PMC4986188

[5] Popovich KJ , Aureden K , Ham DC , et al. SHEA/IDSA/APIC practice recommendation: Strategies to prevent methicillin-resistant *Staphylococcus aureus* transmission and infection in acute-care hospitals: 2022 update. Infect Control Hosp Epidemiol 2023;44:1039–1067. 37381690 10.1017/ice.2023.102 PMC10369222

[6] Nair R , Perencevich EN , Blevins AE , Goto M , Nelson RE , Schweizer ML. Clinical effectiveness of mupirocin for preventing *Staphylococcus aureus* infections in nonsurgical settings: a meta-analysis. Clin Infect Dis 2016;62:618–630. 26503378 10.1093/cid/civ901

[7] Fisher M , Golestaneh L , Allon M , Abreo K , Mokrzycki MH. Prevention of bloodstream infections in patients undergoing hemodialysis. Clin J Am Soc Nephrol 2020;15:132–151. 31806658 10.2215/CJN.06820619 PMC6946076

[8] Tacconelli E , Carmeli Y , Aizer A , Ferreira G , Foreman MG , D’Agata EMC. Mupirocin prophylaxis to prevent *Staphylococcus aureus* infection in patients undergoing fialysis: a meta-analysis. Clin Infect Dis 2003;37:1629–1638. 14689344 10.1086/379715

[9] Pérez-Fontán M , Rosales M , Rodríguez-Carmona A , Falcón TG , Valdés F. Mupirocin resistance after long-term use for *Staphylococcus aureus* colonization in patients undergoing chronic peritoneal dialysis. Am J Kidney Dis 2002;39:337–341. 11840374 10.1053/ajkd.2002.30553

[10] Vasquez JE , Walker ES , Franzus BW , Overbay BK , Reagan DR , Sarubbi FA. The epidemiology of mupirocin resistance among methicillin-resistant *Staphylococcus aureus* at a Veterans’ affairs hospital. Infect Control Hosp Epidemiol 2000;21:459–464. 10926396 10.1086/501788

[11] Smith M , Herwaldt L. Nasal decolonization: what antimicrobials and antiseptics are most effective before surgery and in the ICU. Am J Infect Control 2023;51:A64–A71. 37890955 10.1016/j.ajic.2023.02.004

[12] Miller LG , McKinnell JA , Singh R , et al. 5. The PROTECT trial: a cluster randomized clinical trial of universal decolonization with chlorhexidine and nasal povidone iodine versus standard of care for prevention of infections and hospital readmissions among nursing home residents. Open Forum Infect Dis 2021;8:S4–S5.

[13] McKinnell JA , Singh R , Miller LG , et al. 893. The SHIELD orange county project: a decolonization strategy in 35 hospitals and nursing homes reduces multi-drug-resistant organism (MDRO) prevalence in a Southern California region. Open Forum Infect Dis 2019;6:S23–S24.30895212

[14] Phillips M , Rosenberg A , Shopsin B , et al. Preventing surgical site infections: a randomized, open-label trial of nasal mupirocin ointment and nasal povidone-iodine solution. Infect Control Hosp Epidemiol 2014;35:826–832. 24915210 10.1086/676872 PMC4668802

[15] Anderson MJ , David ML , Scholz M , et al. Efficacy of skin and nasal povidone-iodine preparation against mupirocin-resistant methicillin-resistant *Staphylococcus aureus* and *S. aureus* within the anterior nares. Antimicrob Agents Chemother 2015;59:2765–2773. 25733504 10.1128/AAC.04624-14 PMC4394816

[16] Monstrey SJ , Govaers K , Lejuste P , Lepelletier D , Ribeiro de Oliveira P. Evaluation of the role of povidone-iodine in the prevention of surgical site infections. Surg Open Sci 2023;13:9–17. 37034245 10.1016/j.sopen.2023.03.005 PMC10074992

[17] Maslow J , Hutzler L , Cuff G , Rosenberg A , Phillips M , Bosco J. Patient experience with mupirocin or povidone-iodine nasal decolonization. Orthopedics 2014:37, e576–e581. 24972440 10.3928/01477447-20140528-59

[18] Racila AM , O’Shea AMJ , Nair R , et al. Using nasal povidone-iodine to prevent bloodstream infections and transmission of *Staphylococcus aureus* among haemodialysis patients: a stepped-wedge cluster randomised control trial protocol. BMJ Open 2021;11:e048830–e048830. 10.1136/bmjopen-2021-048830 PMC8647395 34862278

[19] VERBI Software. MAXQDA 2022 [Computer Software]. Berlin, Germany: VERBI Software; 2021.

[20] Garrison DR , Cleveland-Innes M , Koole M , Kappelman J. Revisiting methodological issues in transcript analysis: negotiated coding and reliability. Internet Higher Educ 2006;9:1–8.

[21] Kuckartz U. Qualitative Text Analysis: A Guide to Methods, Practice and Using Software/Udo Kuckartz. Los Angeles: SAGE; 2014.

[22] Khan A , Khan AH , Adnan AS , Sulaiman SAS , Mushtaq S. Prevalence and predictors of depression among hemodialysis patients: a prospective follow-up study. BMC Public Health 2019;19:531–531. 31072378 10.1186/s12889-019-6796-z PMC6507067

[23] Farrokhi F , Abedi N , Beyene J , Kurdyak P , Jassal SV. Association between depression and mortality in patients receiving long-term dialysis: a systematic review and meta-analysis. Am J Kidney Dis 2014;63:623–635. 24183836 10.1053/j.ajkd.2013.08.024

[24] Palmer S , Vecchio M , Craig JC , et al. Prevalence of depression in chronic kidney disease: systematic review and meta-analysis of observational studies. Kidney Int 2013;84:179–191. 23486521 10.1038/ki.2013.77

[25] Jones DJW , Harvey K , Harris JP , Butler LT , Vaux EC. Understanding the impact of haemodialysis on UK national health service patients’ well-being: a qualitative investigation. J Clin Nursing 2018;27:193–204. 10.1111/jocn.13871 PMC6853155 28498615

[26] Alosaimi FD , Asiri M , Alsuwayt S , et al. Psychosocial predictors of nonadherence to medical management among patients on maintenance dialysis. Int J Nephrol Renovascular Dis 2016;9:263–272. 10.2147/IJNRD.S121548 PMC5096770 27826207

[27] Cukor D , Rosenthal DS , Jindal RM , Brown CD , Kimmel PL. Depression is an important contributor to low medication adherence in hemodialyzed patients and transplant recipients. Kidney Int 2009;75:1223–1229. 19242502 10.1038/ki.2009.51

[28] Fernandes Agreli H , Murphy M , Creedon S , Ni Bhuachalla C , O’Brien D , Gould D , et al. Patient involvement in the implementation of infection prevention and control guidelines and associated interventions: a scoping review. BMJ Open 2019;9:e025824–e025824. 10.1136/bmjopen-2018-025824 PMC6475448 30904866

[29] Kim S , Lee HZ. The lived self-care experiences of patients undergoing long-term haemodialysis: a phenomenological study. Int J Environ Res Public Health 2023;20:4690. 36981599 10.3390/ijerph20064690 PMC10048782

[30] See I , Shugart A , Lamb C , Kallen AJ , Patel PR , Sinkowitz-Cochran RL. Infection control and bloodstream infection prevention: the perspective of patients receiving hemodialysis. Nephrol Nursing J: Journal of the American Nephrology Nurses’ Association 2014;41:37–40. PMC4697925 24689263

[31] Kear T , Evans E , Hain D , Schrauf C , Dork L. Patients’ perceptions of hemodialysis catheter care practices at home before and after eliminating a protective dressing and implementing a showering protocol. J Infect Prev 2013;14:208–212.

[32] Miller HM , Tong A , Tunnicliffe DJ , et al. Identifying and integrating patient and caregiver perspectives for clinical practice guidelines on the screening and management of infectious microorganisms in hemodialysis units. Hemodialysis Int 2017;21:213–223. 10.1111/hdi.12457 27389043

[33] Gray NA , Toy L , Dalla-Bona K , Broom J , Gray M. The lived experience of haemodialysis patients managed with transmission-based precautions for MDRO colonisation: a qualitative study. Infection, Dis Health 2022;27:211–218. 10.1016/j.idh.2022.05.003 35690584

[34] Fitzgerald TA , Tyner K , Drake M , Beach S , Rupp M , Schwedhelm S , et al. A needs-assessment survey of healthcare professionals accountable for infection prevention and control in hemodialysis setting. Am J Infect Control 2020;48:S4–S5.

[35] Böhmert M , Kuhnert S , Nienhaus A. Psychological stress and strain in dialysis staff-a systematic review. J Ren Care 2011;37:178–189. 22035362 10.1111/j.1755-6686.2011.00236.x

[36] Pawłowicz-Szlarska, E. , Forycka, J. , Harendarz, K. et al. Organizational support, training and equipment are key determinants of burnout among dialysis healthcare professionals during the COVID-19 pandemic. J Nephrol 2022, 2077–2086. 36040565 10.1007/s40620-022-01418-6 PMC9425824

[37] McAlearney AS , Hefner JL. Facilitating central line–associated bloodstream infection prevention: a qualitative study comparing perspectives of infection control professionals and frontline staff. Am J Infect Control 2014;42:S216–S222. 25239713 10.1016/j.ajic.2014.04.006

[38] Pedersen LL , Pryor R , Bearman G. Healthcare worker perceptions of healthcare-associated infections and health inequity. Antimicrob Stewardship Healthcare Epidemiol 2023;3:e134–e134. 10.1017/ash.2023.196 PMC10428151 37592965

